# Customer Relationship Management Based on SPRINT Classification Algorithm under Data Mining Technology

**DOI:** 10.1155/2022/6170335

**Published:** 2022-04-14

**Authors:** Yazhou Sun, Xueqing Tan

**Affiliations:** ^1^Department of Economic Management, Pingdingshan Polytechnic College, Pingdingshan 467000, Henan, China; ^2^School of Electrical Engineering and Automation, Henan Polytechnic University, Jiaozuo 454003, Henan, China

## Abstract

Under the advance of computational intelligence, customer relationship management system based on data mining technology can not only bring more economic benefits to an enterprise but also improve the management and decision-making level of Chinese enterprises. In this paper, the application of data mining technology in customer relationship management (CRM) is analyzed, and four data mining modes are realized: customer classification, cross-marketing, customer acquisition, and customer retention. In the data mining module, SPRINT classification algorithm is used in customer classification. At the same time, FP-growth, an association rule algorithm without candidate set, is applied in cross-marketing, which enhances the practicability of the system. The algorithm of optimal customer retention strategy under digital intelligence technology is adopted in customer retention, which makes up for the shortcomings of traditional CRM system and helps enterprises to better operate and adjust marketing strategies.

## 1. Introduction

With the rapid development of technology, more and more enterprises find that products tend to be homogeneous in performance, price, appearance, and even advertising. In this environment, the value of customers has become more important, which will also directly affect the value of enterprises. Enterprises in China are facing more and more competition. In the early days, enterprises began to use databases to store customer information and analyze them by computer [1]. To achieve truly scientific customer relationship management, data mining technology is indispensable. The emerging data mining technology in recent years can provide technical support for customer relationship management, better maintain customer relationship, and give full play to the role of customer management system [2].

Customer relationship management system based on data mining technology has been applied to many industries, such as banks, large-scale retail industries, and e-commerce companies [3]. Through the application of data mining technology in customer relationship management system, credit evaluation, customer retention, price setting, financial analysis, and marketing planning can be carried out in these industries, thereby reducing costs and increasing profits for enterprises. Customer relationship management system based on data mining technology can not only bring more economic benefits to an enterprise but also improve the management and decision-making level of Chinese enterprises.

## 2. Overview of Customer Information Management and Data Mining Technology

### 2.1. Customer Information Management

#### 2.1.1. Concept and Origin

The idea of customer relationship management (CRM) comes from the marketing theory, management science, behavioral relationship research, and other disciplines of western enterprises. With the arrival of the industrialized society, the productive forces have been greatly developed. After automation has gradually entered the industrial field, there are more types of products, and the demands of consumers become diversified. In addition, the rapid progress of technology has also broadened consumers' choices. The technology-oriented or product-oriented seller's market in the past gradually became a customer-oriented buyer's market. In order to cope with the new changes, maximize the profits of enterprises, and occupy more markets, the marketing methods of enterprises have also changed. Especially after entering the postindustrial era, knowledge will become the dominant force [4]. With the development of modern information technology, enterprises can survive in the fierce market competition by capturing customers' needs through technical means combined with traditional theories and providing targeted products and services to meet customers' real needs [5]. Therefore, the theory and practice of CRM have been greatly developed.

Customer relationship management can be divided into three phrases: customer, relationship, and management.

1. *Customers*. The customers here do not just refer to “people” who use products or services in a narrow sense. They can be an organization, a person, or a group. Nor is it just the user of the final product or service. Generalized customers can be any organization or individual, as long as it is related to the operation of the enterprise. For example, employees in an enterprise are internal customers of the enterprise, and the department of the next process is the customer of the last process. Externally, customers can also be dealers, trade associations, and so on.
2. *Relationship*. Relationship refers to the behavior and feeling state between enterprises and customers. Behavior mode is the performance of the degree of relationship in behavior, such as repeated purchase or reduced purchase frequency. Feeling refers to the expression of the degree of relationship in attitude, which can include preference or aversion, spreading good, or bad word of mouth [6]. In customer relationship management, relationship is the link between enterprise customers and the core purpose of the whole customer relationship management.
3. *Management*. Management is more of a means. Although customer relationship management was put forward only in the 1980s, its customer-centered concept has been widely used in business activities for a long time. With the advent of the computer era, customer relationship management can be realized more easily. Through various modern tools, the resources of enterprises can be conveniently organized. Adjust, organize, or allocate resources according to the state of customer relationship [7].

#### 2.1.2. Classification and Composition

**Figure 1:**
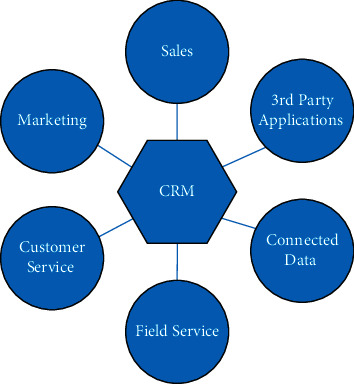
Organizational structure of CMR.

Customer relationship management (CRM) is a systematic concept, which includes CRM strategy, CRM system, and CRM marketing strategy, all of which are indispensable. Its main goal is to optimize the management of customer data and expect more customization for the ever-changing customer demand [8]. Customers who request customer service through the website should be recognized and provide personalized responses to their requests. CRM and cloud computing are technologies aimed at centralized customer data management because it allows companies to respond effectively to customer requests [9]. At the same time, big data depends on using the massive data generated by CRM channels to improve knowledge. Therefore, CRM solutions will mainly involve business teams (e.g., marketing) as well as marketing teams, IT teams, or customer service teams (Figure 1). Therefore, the centralized information in CRM will be shared by all relevant teams to ensure the best customer relationship management.

**Figure 2:**
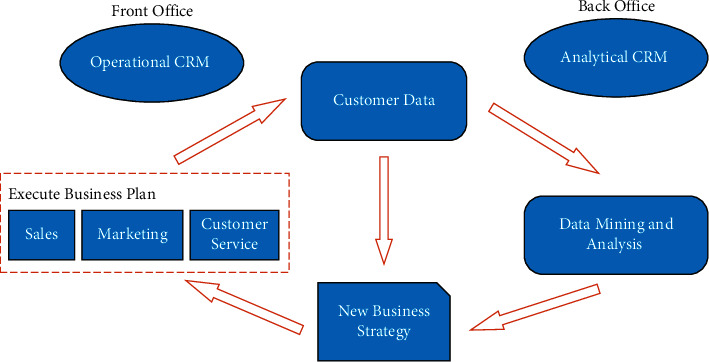
Relationship between different types of CRM.

According to the activities and nature of work, CRM systems are generally divided into three categories: operational CRM, cooperative CRM, and analytical CRM [10]. CRM in operation makes marketing automation, thus generating clues of contact in CRM and interdepartmental collaboration to share data to achieve strategic goals and objectives. However, analysts are responsible for enhancing business status according to decisions provided by customer data.  Figure 2 shows the integration of operational and analytical CRM with data mining and business planning. In today's business world, new technologies enable organizations to more accurately target individual customers or market segments [11]. At the same time, advanced marketing technology enables people to pay more attention to the customer-centered point of view.

### 2.2. Data Mining Technology

#### 2.2.1. Process of Data Mining

**Figure 3:**
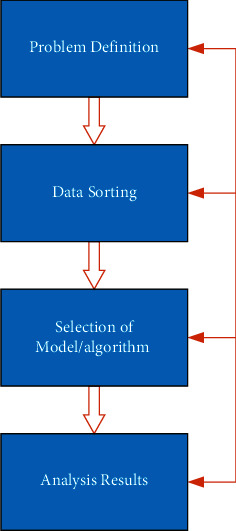
Data mining process.

Data mining technology itself is the core of knowledge discovery. It needs to sort out hidden and valuable knowledge from a large amount of data. In the aspect of marketing decision-making, it is necessary to analyze the data of enterprises, discover the hidden rules, and then further model them. Generally speaking, data mining has four processes [12–14], problem definition, data sorting, selection of the model/algorithm, and analysis results, as shown in Figure 3.

The process of data mining is not one-way, but a cycle. After the results are formed, there may be errors or irrelevant parts in them. Irrelevant parts represent the abnormal data, which can't be used to the following algorithms, reselect the algorithm/model, or adjust the parameters to exclude irrelevant parts. If that result is found to deviate greatly from the target, it may even need to go back to the first step and reestablish goals. Therefore, data mining is a dynamic and cyclic process. Complete data mining is a process of continuous feedback.

#### 2.2.2. General Algorithms

According to the different objectives of data mining or different data types, there are many algorithms currently used in the field of data mining [15–17]: Bayesian algorithm, decision tree, clustering algorithm, association algorithm, neural network algorithm, regression algorithm, association rule algorithm, and so on:

1. Bayesian algorithm is an algorithm commonly used for classification prediction, which is based on Bayesian theory and uses statistics to classify samples. The Bayesian network is constructed by statistical data, and samples of unknown categories are classified and predicted according to the Bayesian network. Then, the possibility that samples belong to each classification is predicted and the most probable classification is selected [17]. Compared with other algorithms, Bayesian has the advantages of less computation and higher accuracy. Even the simplest Naive Bayes algorithm can get an excellent result which is especially suitable for the task of sample classification and prediction.
2. Decision tree is a regression algorithm, which uses recursion to establish a tree-like structure of classification rule. Firstly, the algorithm finds the input attribute that has the greatest influence on the target variable from the sample data and establishes the root node. According to the different values of input attributes, the sample data are divided into different subsets, and then the subsets are gradually divided according to the degree of influence between them and the input attributes until all attributes are included in the tree structure or the splitting is stopped because of insufficient subset samples. Finally, a tree structure is formed. Decision tree can be used to examine the influence of input attributes on target variables. Decision tree is commonly used to classify samples according to attributes. It can also be used to predict unknown classified samples [18]. The advantage of decision tree is that users do not need to have a deep understanding of the attributes of samples, and it can learn by itself and discover rules according to the sample data.
3. Clustering algorithm is also an algorithm for sample classification. Multidimensional space is established according to various attributes of samples, and classes are sorted according to the geometric distance between samples in the multidimensional space. Unlike other classification algorithms, clustering algorithms do not need to know the classes to be divided beforehand, and the formation of the class is completely generated automatically. The advantage of the clustering algorithm is that it hardly needs any prior knowledge, which is an unsupervised algorithm.
4. Neural network algorithm is an algorithm that imitates the characteristics of connections in brain neuron and is often used for classification. Generally speaking, the neural network has three levels: input, optional hiding, and output. Each neuron receives one or more inputs and then produces one or more identical outputs according to a simple nonlinear function. The sample is input into the hidden layer from the input layer and finally to the output layer. Neurons at the same level are not connected [19]. The advantages of the neural network are the ability to self-learn; realization of the association function [20]; multichannel parallel computing, which can be used for particularly complex problems.
5. The commonly used linear regression algorithm is to find the linear function between dependent variable and independent variable according to statistical data. Regression algorithm can find the correlation between independent variables and dependent variables very directly [21], which is often used to forecast marketing, and so on. Time series algorithm is also a kind of regression algorithm, which is to find the relationship between dependent variables, time series, and other possible independent variables, which is often used to predict marketing.
6. Association rule algorithm is used to find the connection or correlation between different sets in data samples. For example, if the customer has a higher probability of buying commodity B after purchasing commodity A, then there is a certain degree of connection between commodity A and commodity B. Association rules allow users to analyze the behavior of customers, which is beneficial to formulate marketing strategies.

### 2.3. Data Mining-Based CMR

Data mining technology has been widely used in all aspects of customer relationship management, such as analyzing the factors that affect customer satisfaction, subdividing customer market, predicting customer behavior, predicting marketing trends, and cross-marketing. Data mining has also brought CMR to a new level.

#### 2.3.1. Architecture Mode

**Figure 4:**
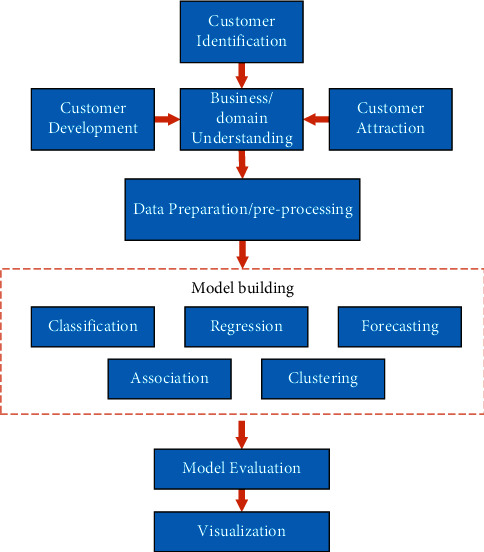
Architecture of the CMR system based on data mining.

- Centralized mode
- The earliest centralized system consists of mainframe and many computer terminals.
- C/S mode
- That is, the client-server model consists of two-tier architecture, in which the server is responsible for data processing and users can obtain graphical interfaces. Servers generally use high-configuration personal computers, workstations, or minicomputers and use large database systems, such as Oracle and SQL Server. Clients need to install dedicated software [22].
- B/S mode
- That is, the browser server mode is an improvement of the C/S architecture where the client only needs a single computer with Internet access. B/S mode is divided into two types [23]. One is that customers need to download special controls and then operate all programs through browsers, which are usually used on intranet [24]. There is also a control that does not need to be downloaded and completely run on the Internet and intranet [25].
- Based on the framework above, close study and management of customer relationships and their interactions will help to identify, attract, and retain effective customers in this field. In the next stage of data preparation or preprocessing, data is prepared for further establishment and evaluation through cleaning, attribute selection, data conversion, and other processes. The model built in the CRM framework is an important step to establish an effective model to meet business needs. These models help to predict customer behavior and evaluate and visualize the effectiveness of measurement models to improve their performance which is shown in Figure 4.

#### 2.3.2. Application Status

At present, in the process of marketing goods and services, enterprises cannot continue to treat them equally as before. Because different types of customers have different preferences, they must provide different marketing strategies to improve customers' satisfaction and loyalty and finally achieve the purpose of profitability by realizing customer value:

1. Customer classification
2. Customer segmentation is the basis of targeted marketing strategy. The category of the customer will be expressed by the customer's own attributes and the customer's purchasing behavior pattern. After mastering a certain amount of customer data, managers can classify existing customers by analyzing their behavior patterns and attributes [26]. Although the loss of customers has caused losses to enterprises, the loss of customer data is also a very valuable asset for enterprises. Through data mining, customers who are easy to lose can be classified. Different types of plans for customer retention can be introduced for different types of easy-to-drain customers, thereby reducing the customer churn rate of enterprises.
3. Forecast of marketing amount
4. By mining the historical data of marketing, the regular behavior of sales can be obtained. For example, by introducing time series, the trend of marketing or whether there is a seasonal change rule can be discovered. Through predictive marketing, managers can better serve customers by adjusting inventory and production capacity, preparing raw materials, and reducing delivery time. Regression analysis can also be used in the management of products' life cycle. With more accurate help to enterprises to determine where products are located and through product strategies of different life cycles, customer loyalty can be improved.
5. Customer churn was found
6. Real customer churn generally does not show clear notice from customers. It is just that customers gradually disappear and do not come back to continue purchasing [27]. However, every purchase of customers will leave its mark of characteristics. Therefore, by analyzing these marks, managers can get the rules of customer churn and find out which customers are going to be lost so that they can modify customer strategies and retain valuable customers as much as possible.

## 3. Design of the CMR System in Marketing Strategy Based on Data Mining

### 3.1. Module Design

In this paper, the CMR system in marketing strategy consists of the following three subsystems [28]: business operation system, customer cooperation system, and data analysis system. Among them, the customer operation subsystem and data analysis subsystem are the most important subsystems, including customer information management and information analysis and processing:

1. Business operation subsystem
2. Mainly with the help of computer technology, it manages all aspects of marketing, sales, and service. The operating system is also known as an invoicing system which can enable enterprises to adopt better methods to achieve optimal results.
3. Customer cooperation subsystem
4. This subsystem includes customer information entry, customer information processing, code management, customer management, supplier management, department management, employee management, product management, and partner management. It mainly manages the interaction between enterprises and customers, including e-mail, customer service center, call center, and electronic community. Putting these together, it means all channels for enterprises interact with customers.
5. Data analysis subsystem
6. By processing and analyzing all kinds of data, using data mining technology to realize customer relationship management, managers can get meaningful information from it. Data information obtained by different ways, such as customer cooperation system and business operating system, should be sorted and summarized so as to help enterprises understand the classification, satisfaction, demand information, and other pieces of useful information of customers.

## 4. Realization of the CMR System in the Marketing Strategy Based on Data Mining

### 4.1. Overall Architecture

**Figure 5:**
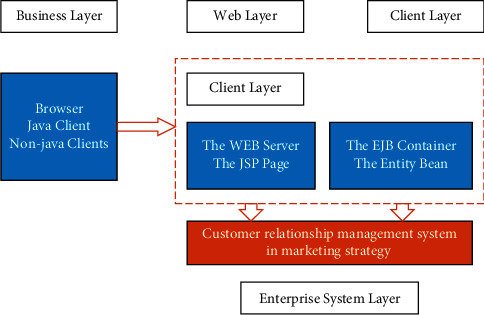
Overall architecture mode of CMR.

The CRM system of shopping malls based on data mining adopts a modular design. Considering the need for modular development and maintenance, the system adopts J2EE architecture and B/S system architecture based on the browser. Its development tool is JBuilderX, the back-end database is Oracle9i, and the application server is implemented by WebLogic8.1.x. The overall architecture is shown in Figure 5.

Data mining-based CRM system in marketing strategy adopts EJB container for data mining. EJB is a stateful session bean, which mainly includes the following six aspects: data selection, customer classification, cross-selling, customer acquisition, customer retention, and result output. The design scheme is as follows:

1. Data input ()
2. In the whole database, make a reasonable judgment on the data submitted by the client to find out more complete and consistent data.
3. Customer segmentation ()
4. According to the different attributes of each customer, customers are divided into different categories according to different classification standards.
5. Cross buying ()
6. Through the buying behavior of customers in a certain period of time, the influence of one commodity on other commodities and whether it is suitable for bundling can be analyzed.
7. Customer obtainer ()
8. By analyzing the customer's response to market activities, some attributes of potential customers can be found.
9. Customer retention ()
10. Establish a customer classification model with churn rate, analyze the churn of a customer, and determine how to keep the customer according to its attribute characteristics.
11. Result output ()
12. Pass the results of data mining back to the client. Include customer information data, marketing campaign data, and customer transaction data. Customer EJB is used as the entity bean to describe the customer information of the system. Session Bean--DataminingEJB EJB is used to wrap entity Bean. CustomerEJB client calls entity beans by interacting with session beans.

The code that defines the remote interface is as follows:

/^*∗*^Dataminigjava^*∗*^/

import java.util.^*∗*^

import javax.ejb.EJBObject.

import java.rmi.RemoteException.

public interface Datamining extends EJBObject.

{

public Boolean dataInput(String selectString) throws.

RemoteException.

public void customerSegmentation(String tablename) throws.

RemoteExecption.

public void crossBuying(String tablename) throws.

RemoteExecption.

public void customerObtainer(String tablename) throws.

RemoteExecption.

public void customerRetention(String tablename) throws.

RemoteExecption.

public String resultOutput(String selectString) throws.

RemoteException.

}

The code for calling the entity Bean in the session Bean is as follows:

public Collection getAllData()

{

Vector vectUserInfos = new Vector();

Try.

{

Contextc tx = new InitialContext();

Object obj = ctx.lookup(“CustomerHome”);

CustomerHome customerHome=(CustomerHome).

PortableRemotcObject.narrow(obj,CustomerHome.class).

Collection collection = customerHome.findAll();

Return collection;

}

catch (Exception e).

{

e.printStackTrace();

}

}

### 4.2. Algorithm Design

Based on the above research conclusions, the decision tree algorithm is adopted for customer classification. Association rule algorithm is adopted for cross-selling. Classification algorithm is adopted for customer acquisition; The best customer retention strategy algorithm is adopted for customer retention. The algorithm design of these data mining technologies is analyzed in the following.

#### 4.2.1. Data Mining Algorithm for Customer Classification

In the formulation of marketing strategy, the classification of customer information is relatively simple, and SPRINT algorithm in decision tree algorithm is adopted here. The hash table used in it does not need to reside in memory, so it can handle larger training data sets and classify them more accurately. Enterprises can formulate one-to-one marketing strategies according to different types of customers. The specific algorithm process is given in Algorithm 1.

Establish different attribute lists according to each field in the customer information table, and presort them. For example, in the list of age attributes, it is sorted by age from large to small. Then find out the best splitting point of each node in the decision tree. First, the root node is created, then the candidate split points are selected as 15,23,29, and their Gini values are calculated. Select the smallest as the best splitting point of the attribute list. By calculation, the best split point is 29, which divides the age attribute list into two parts. After that, the decision tree is divided into two branches: the left branch is the data record whose age is less than 29, and the right branch is the data record whose age is greater than or equal to 29. Then a hash table is established according to the root node, recording which child node each data belongs to, respectively. Finally, continue to take the above method to calculate the best splitting points for the two branches created and stop when the data in the attribute list are of the same class or the number of data is very small.

#### 4.2.2. Data Mining Algorithm for Cross-Selling

According to the customers' purchasing, establish the database of customers' transaction data, and then conduct data mining on these data to find out which commodities are usually purchased together. Choose the transaction records of purchasing more than 5 kinds of goods in the mall; each transaction record includes the transaction number, purchase time, and purchased products. The transaction data of these customers are shown in Table 2.

Among the association rule algorithms, Apriori classical algorithm is an association rule algorithm based on mining sets in customer transaction database [30]. The core method is recursion, that is, the method based on frequency set. However, there are some shortcomings when Apriori classical algorithm is introduced into the cross-selling of CRM system. When analyzing the transaction data, the candidate set is always very large, which will seriously affect the efficiency and may make the system abnormal.

FP-growth algorithm is an improvement of the Apriori algorithm, and it is an association rule algorithm [31] that does not need candidate sets. Therefore, the FP-growth algorithm is selected to solve the problem of cross-selling. In this way, through the data mining technology, when making the publicity plan, the managers can distribute the publicity materials of related product at the same time or match products for customers according to these association rules.

#### 4.2.3. Data Mining Algorithm for Customer Retention

The target variable includes customer attributes, service attributes, and customer consumption data. According to the customer classification model with churn rate, which classification each customer should belong to is determined.

According to the customer classification model with churn rate, the relevant managers can discover the customers that may be lost. If the possibility of customer churn is high, the company should adopt various promotional measures to improve customer loyalty so as to reduce customer churn and customer churn rate. To establish such a customer classification model, it is necessary to calculate the loss rate of each classification. Churn rate of classified customers is calculated by dividing the number of customers churned by the total number of classified customers.

The optimal customer retention strategy is to establish a simple decision tree of customer classification with churn rate. According to different customer attributes such as service type, gender, and credibility, each leaf node represents a customer category with different values. For example, the first leaf node represents a female customer, and the wastage rate is 0.1. The second leaf node represents male customers, and the wastage rate is 0.8. The third leaf node represents general customers, and the wastage rate is 0.2. The fourth leaf node represents loyal customers, and the customer churn rate is 0.5.

The algorithm of the best customer retention strategy can calculate the node that brings the highest net profit to the company. Finding the best node is the best strategy for customer retention. Its specific algorithm is given in Algorithm 4.

## 5. Conclusion

Data mining technology has been applied in customer relationship management system, which can make enterprises better understand customers and make better business strategies, thus improving the quality of marketing decisions. In this paper, the technology of data mining with CRM in marketing strategy is combined, and data processing methods are provided. In addition, customer classification, cross-selling, customer acquisition, and customer retention are realized in the data mining module. The SPRINT classification algorithm is used in customer classification, which improves the accuracy of customer classification, thus making the relevant decisions of companies more credible. At the same time, FP-growth, an association rule algorithm without candidate set, is applied in cross-selling, which makes the system more practical. The algorithm of the optimal customer retention strategy is adopted in customer retention so as to help the companies to make decisions, thus better retaining customers and making the store get the maximum profit.

## Figures and Tables

**Algorithm 1:**
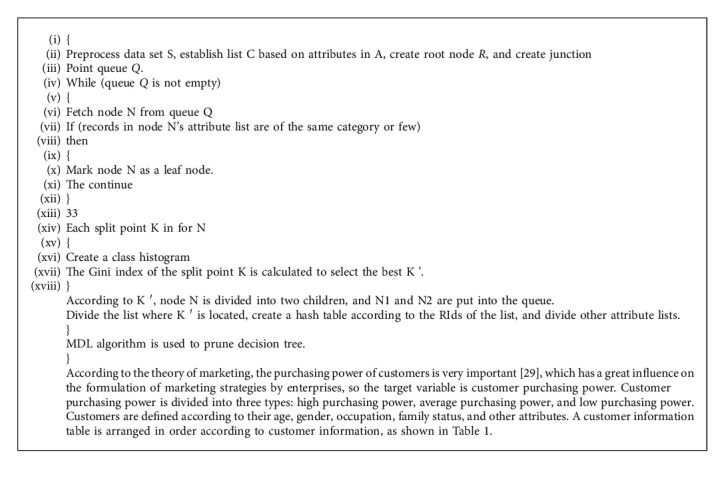
SPRINT algorithm

**Algorithm 2:**
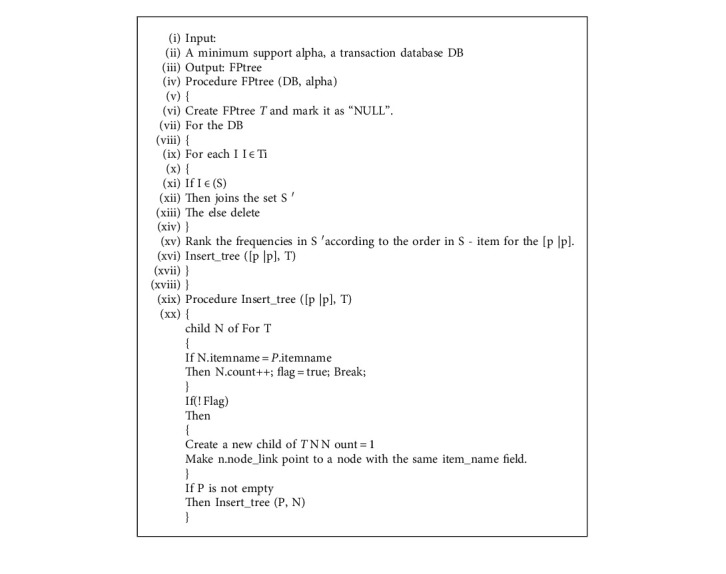
FPtree based on database.

**Algorithm 3:**
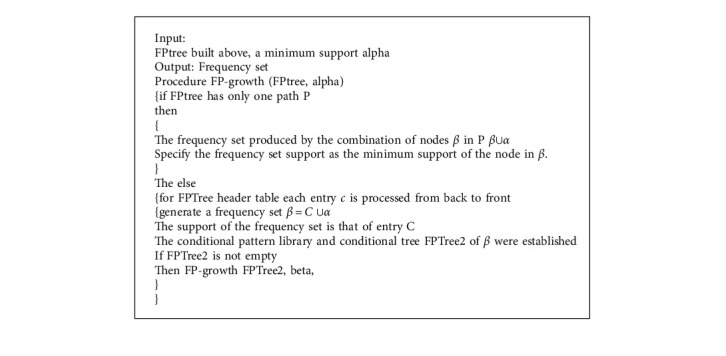
Generation of frequent sets.

**Algorithm 4:**
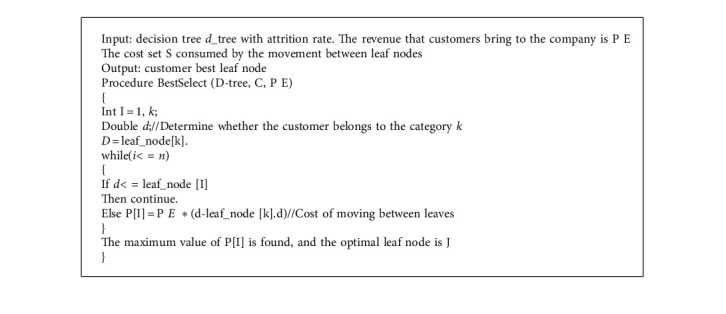
Optimal customer retention strategy algorithm.

**Table 1:** Basic information of customers.

Id	Age	Gender	Income	Family status	Professional category	Purchasing power
ID902310	18	F	21378.2	General	1	Low
ID902311	26	M	12892.3	Good	3	High
ID902312	20	F	5656.2	General	1	Low
ID902313	24	F	7998.2	Good	3	High
ID902314	16	M	56569.6	Poor	2	High

**Table 2:** Customer transaction records.

Transaction number	Time	Product
0000001	20-6-18	F118,A003,C151,D027,G055,I328,M045,P147
0000002	20-6-18	F118,A150,B013,F051,F027,G055,H028,L025
0000003	20-6-18	A003,B003,F028,M102,G023
0000004	20-6-18	A003,B203,C151,F118,L122,M045,O057
0000005	20-6-18	B023,F118,H025,J015,O057
0000006	20-6-18	A003,B203,C151,F118,L122,M045,O057
0000007	20-6-18	F118,A150,B013,F051,F027,G055,H028,L025
……		
000000500	20-6-18	F033,0018,B021,F006,L012,F145,E245

## Data Availability

The dataset can be accessed upon request.
